# Behavioural Classification of Cattle Using Neck-Mounted Accelerometer-Equipped Collars

**DOI:** 10.3390/s22062323

**Published:** 2022-03-17

**Authors:** Dejan Pavlovic, Mikolaj Czerkawski, Christopher Davison, Oskar Marko, Craig Michie, Robert Atkinson, Vladimir Crnojevic, Ivan Andonovic, Vladimir Rajovic, Goran Kvascev, Christos Tachtatzis

**Affiliations:** 1 BioSense Institute, 21101 Novi Sad, Serbia; oskar.marko@biosense.rs (O.M.); crnojevic@biosense.rs (V.C.); 2 Department of Electronic and Electrical Engineering, University of Strathclyde, Glasgow G1 1RD, UK; mikolaj.czerkawski@strath.ac.uk (M.C.); christopher.davison@strath.ac.uk (C.D.); c.michie@strath.ac.uk (C.M.); robert.atkinson@strath.ac.uk (R.A.); i.andonovic@strath.ac.uk (I.A.); christos.tachtatzis@strath.ac.uk (C.T.); 3 School of Electrical Engineering, University of Belgrade, 11000 Belgrade, Serbia; rajo@etf.rs (V.R.); kvascev@etf.bg.ac.rs (G.K.)

**Keywords:** precision agriculture, cattle behaviour monitoring, feature selection

## Abstract

Monitoring and classification of dairy cattle behaviours is essential for optimising milk yields. Early detection of illness, days before the critical conditions occur, together with automatic detection of the onset of oestrus cycles is crucial for obviating prolonged cattle treatments and improving the pregnancy rates. Accelerometer-based sensor systems are becoming increasingly popular, as they are automatically providing information about key cattle behaviours such as the level of restlessness and the time spent ruminating and eating, proxy measurements that indicate the onset of heat events and overall welfare, at an individual animal level. This paper reports on an approach to the development of algorithms that classify key cattle states based on a systematic dimensionality reduction process through two feature selection techniques. These are based on Mutual Information and Backward Feature Elimination and applied on knowledge-specific and generic time-series extracted from raw accelerometer data. The extracted features are then used to train classification models based on a Hidden Markov Model, Linear Discriminant Analysis and Partial Least Squares Discriminant Analysis. The proposed feature engineering methodology permits model deployment within the computing and memory restrictions imposed by operational settings. The models were based on measurement data from 18 steers, each animal equipped with an accelerometer-based neck-mounted collar and muzzle-mounted halter, the latter providing the truthing data. A total of 42 time-series features were initially extracted and the trade-off between model performance, computational complexity and memory footprint was explored. Results show that the classification model that best balances performance and computation complexity is based on Linear Discriminant Analysis using features selected through Backward Feature Elimination. The final model requires 1.83 ± 1.00 ms to perform feature extraction with 0.05 ± 0.01 ms for inference with an overall balanced accuracy of 0.83.

## 1. Introduction

Autonomous cattle behaviour monitoring systems have grown in importance over the recent past. Sensor-based technologies are now starting to be accepted as an enhancement to traditional visual inspection, the latter being both time-consuming and labour-intensive. In the UK, there has been a steady decline in the number of milk producers, whilst at the same time the average size per herd has risen as small-scale farm holdings have departed the industry sector due to the economic pressure. The average number of cows per herd has also grown from ~75 in 1996 to ~155 in 2020 [1]; and during the same period, milk production has increased marginally, from ~13 M litres in 2008 to ~15 M litres in 2020 [1]. As a direct consequence, the time available to observe herds has reduced significantly, with farmers now more amenable to relying on technology-based systems for extensive monitoring [2,3].

Systems such as neck-mounted collars, leg and ear tags that monitor dairy and beef cattle are now enjoying increased adoption. Such systems provide early information on health and welfare issues, and identify the onset of oestrus, both of which form the basis for a decision support system that advises farmers on the most appropriate interventions that enhance the efficiency of current practices [4,5,6].

In this paper, the use of a neck-mounted accelerometer-based collar to identify eating and rumination signatures is reported. A muzzle-mounted halter pressure sensor was used in order to collect the ground truth data. The halter has proved to yield high correlation between identified and visually observed behaviours and has become a widely accepted means of gathering ground truth data throughout the precision livestock community. A study by [7] compared halter-based labels and video annotations and reported an F1 Score of 0.932 for rumination. Additionally, a high Spearman correlation of 0.96 and 0.75 for rumination, and 0.96 and 0.81 for eating, respectively, was reported in [8,9]. Three classification algorithms are considered here and a comparison of their ability to discriminate different cattle states has been performed. Data from 18 steers were acquired during three farm trials in the United Kingdom (Easter Howgate Farm, Edinburgh, UK). A total of 42 features were initially extracted from the data, followed by a systematic reduction in dimensionality to decrease model complexity, easing the transformation of the raw sensor data into actionable information and optimising the trade-off between model performance, computation complexity and memory footprint.

The paper is organised as follows. Section 1 represents a brief introduction and Section 2 provides a summary of related work. Section 3 presents a short description of the data acquisition methodology. Section 4 describes the adopted methodology and details the dimensionality reduction methods, while Section 5 describes the classification algorithms considered. Section 6 evaluates the accuracy of the classifications and the efficiency of implementation of the proposed approaches. Section 7 draws conclusions and summarises key findings. The full range of feature definitions are given in the Appendix A.

## 2. Related Work

A range of solutions for cattle behaviour identification have been reported, many based on classical Machine Learning (ML) algorithms [10,11,12,13,14,15,16,17], but the recent adaption of Deep Learning (DL) techniques has significantly increased the potential to optimise the efficiency of artificial intelligence enabled classification solutions [18,19,20,21].

Convolutional Neural Networks (CNNs) have been used for classification of grazing and non-grazing periods [18]; given the output is binary, the development is less demanding compared to multi-state behavioural classification. A highly accurate performance classifier based on a 3-axis accelerometer/gyroscope/magnetometer data and a Recurrent Neural Network with Long Short-Term Memory (RNN-LSTM) able to identify 8 cattle behaviours has been reported in [19]. Although the RNN-LSTM algorithm achieved accurate cattle behaviour classification, its operational deployment on low-cost, low-power processors is prohibitively challenging due to significant model complexity. The approach which overcomes the operational implementation challenges of complex Deep Learning (DL) models was implemented through an iterative structured pruning process in [21]. The results confirm that the CNN architecture can be supported on low-power micro-controllers with an operational lifetime of 5.7 years. The methodology achieved a model compression of 14.30 with minimal loss of performance; however, additional effort to create the approach that overcomes the implementation challenges is required.

In most instances, although classical ML algorithms do not require model reduction, a further decrease in computational complexity and memory footprint requirement will enhance device efficiency and prolong battery lifetime. An approach [12] based on Decision Tree (DT) and Support Vector Machine (SVM) algorithms, using data from neck-mounted collars sampled at 10 Hz, demonstrated high performance classification for three cattle states viz. ‘eating’, ‘rumination’ and ‘other’. The overall accuracy, validated by human observation, was 0.90 and 0.93 using DT and SVM algorithms, the latter classifying ‘eating’ and ‘rumination’ with a precision of 0.92 and 0.88 and sensitivity of 0.85 and 0.92, respectively. Data were acquired from 10 animals over a period of 5 days giving a total monitoring time of 60 h. A similar study also demonstrated the use of a SVM to identify a larger number of cattle states including ‘eating’, ‘rumination’, ‘standing’, ‘lying’ and ‘walking’ [11] using accelerometer measurement data sampled at 10 Hz from 30 animals. The approach produced results with a precision of 0.78 ± 0.01, with ‘eating’ and ‘rumination’ classified with a precision of 0.81 ± 0.03 and 0.86 ± 0.02 and sensitivity of 0.75 ± 0.04 and 0.75 ± 0.02, respectively. The classification accuracy of both states was reported to be 0.96 ± 0.01 and 0.92 ± 0.01. Ground truth data, obtained through both direct animal observation and video annotation, provided a highly appropriate validation dataset; nevertheless, owing to the significant effort required, a relatively small dataset of 95.5 h in total was acquired.

The present paper advances the state-of-the-art in several areas: It proposes a methodology to systematically reduce the dimensionality using a number of feature selection techniques and, coupled with appropriate ML algorithms, to deliver accurate identification of ‘eating’, ‘rumination’ and ‘other’ cattle behaviours using data from 3-axis accelerometer neck-mounted collars. The development harnesses a comparable dataset size to other reported studies in terms of the number of animals, but the total number of observation hours is significantly higher. The studies conducted in [11,12] proposed the use of 28 and 16 features, respectively, derived from the raw accelerometer data; however, the motivation for selecting the corresponding number of features and the features themselves was not directly specified. The methodology reported here begins with 42 knowledge-specific and generic time-series features and follows a systematic feature reduction process, resulting in 7 features that yield near optimum classification performance while maintaining low model complexity. As most datasets are not publicly available, a comparison of the classification performance of the proposed model with the data used to develop other algorithms was not possible. The data underpinning the current study have been made publicly accessible to stimulate the creation of new algorithms and permit the community to perform direct comparisons.

## 3. Data

**Figure 1:**
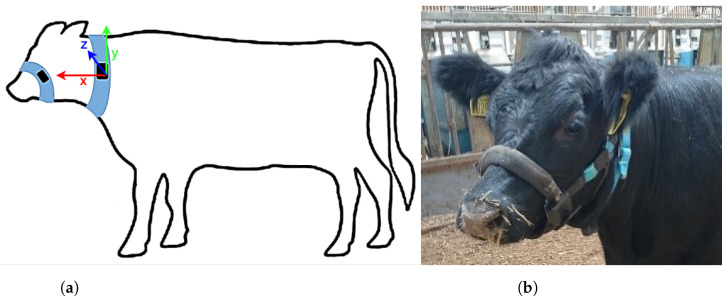
Placement of a RumiWatch muzzle-mounted halter and Afimilk Silent Herdsman neck-mounted collar. (**a**) Axis orientation diagram. (**b**) Photograph illustrating sensor placement.

The cattle were housed indoors in a straw setting and fed a Total Mixed Ration (TMR) ad libitum. Data, collected during three farm trials in the United Kingdom (Easter Howgate Farm, Edinburgh, UK) were acquired from a total of 18 Limousin Cross-Breed steers equipped with Afimilk Silent Herdsman [5] neck-mounted collars and Rumiwatch halters [22] mounted on the muzzle (Figure 1). The collar comprised a 3-axis accelerometer sampled at 10 Hz with range of ±2 g and 12-bit resolution, an SD card for storage, and a Real Time Clock (RTC). The halter consisted of a pressure sensor, an SD card and RTC producing behaviour classification at frequency of 10 Hz. The SD cards from both systems were collected and the recordings with total duration of 3460 h were verified for time alignment (the dataset is publicly available at https://www.doi.org/10.5281/zenodo.4064802, accessed on 16 February 2022).

The collars provided acceleration values orientated in x-, y- and z-directions, i.e., parallel, vertical and perpendicular to the body of the animal, capturing both head and neck muscle motions. The halter, through pressure changes induced by movements of the jaw, provided the ground truth of the following animal states:

- Eating—the animal is ingesting food.
- Rumination—the animal is regurgitating to further breakdown ingested food and improve nutrient absorption.
- Other—the animal is engaged in an activity which is neither ruminating or eating.

### Data Preparation

At the outset, both the accelerometer and halter time sequences were segmented into 90 s blocks [10,12,19], with each block of the accelerometer signal assigned to only one behaviour state for truthing. The acceleration in y-direction—oriented vertical to the animal body i.e., perpendicular to the ground is the one that captures both head and neck muscle motions, central to the identification of the target cattle states; for that reason, only y-axis data was used for analysis [15]. Considering that the halter provides measurements at a frequency of 10 Hz and that there are instances of more than one cattle behaviour during the 90 s period, a majority vote was applied within each block to indicate the primary behaviour.

## 4. Model Design

**Figure 2:**
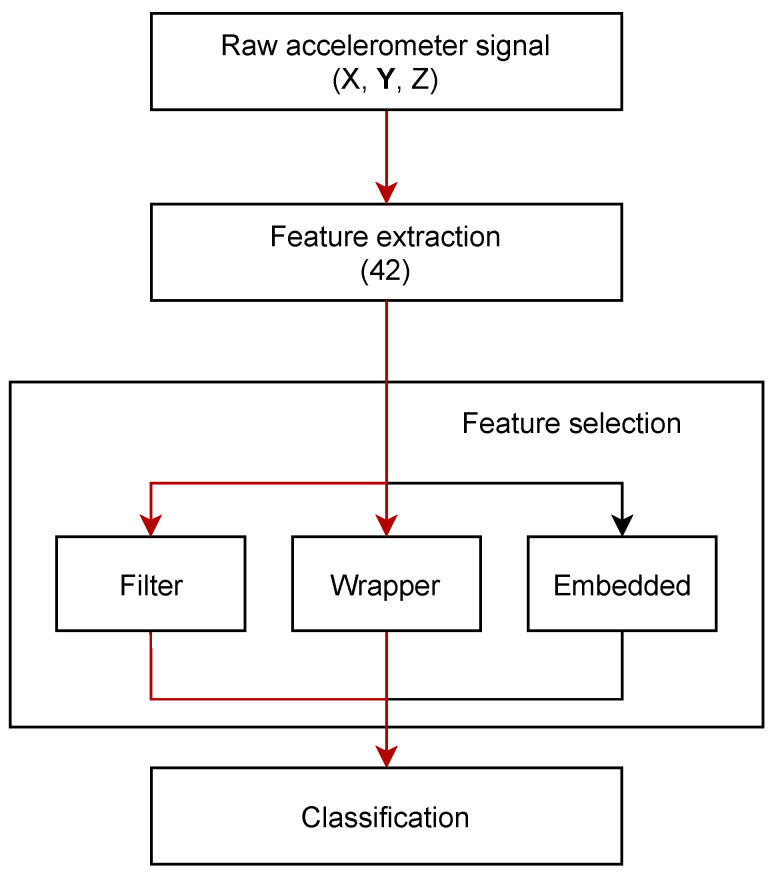
A block diagram showing the methodology starting from the raw data to training and evaluation of the classification algorithms. The red arrows indicate the adopted methodology followed in this work.

A total of 42 features, defined in the Appendix A (Table A1), were extracted from raw accelerometer signals for each of the 90 s blocks as the basis for the discrimination between cattle behaviours. All features used within the analysis are derived using the *tsfresh* Python package [23] with the exception of two knowledge-specific features; *FFT amplitude* in the band 2–4 Hz and *Spectral flatness*. Specific features were selected, informed by the knowledge that the dominant frequency of the rumination motion is centered around ~3 Hz and manifests as a significant spectral peak, while the eating frequency content is spread over a wider band, characterised by a relatively flat spectrum. Given the relatively high number of extracted features, the performance of the classification model is compromised due to the curse of dimensionality. A highly dimensional feature space also has ramifications in respect of increased computation complexity and memory footprint hindering the ability to deploy low-cost, low-power on-farm implementations. Therefore, a systematic reduction of features was performed in order to decrease model complexity but not at the expense of a reduction in discrimination performance between three cattle states of ‘eating’, ‘rumination’ and ‘other’. The reduction phase is followed by evaluation of three classification algorithms, namely, Hidden Markov Model (HMM), Linear Discriminant Analysis (LDA) and Partial Least Squares Discriminant Analysis (PLS-DA). A schematic of the end-to-end development pipeline is illustrated in Figure 2, the red arrows representing the applied process flow, while the black arrows illustrate an alternative, relevant methodology not considered here. All components of the adopted methodology presented within the block diagram are further analysed in more detail.

### 4.1. Training and Validation

Three steers from the total of 18, each drawn from a distinct farm trial, were randomly selected to form a dataset prior to any pre-processing. The data from the three steers are used at the final stage only in order to evaluate the methodology and are not considered in the dimensionality reduction process nor in the training of the classification model. The remaining 15 steers are used to optimise the combination of features and classification model parameters through a 5-fold cross-validation process; twelve steers are used as the training set, with the remaining three forming the validation set. The cross-validation process is repeated 5 times so that each steer is present in the validation set precisely once. Further, the complete 5-fold cross-validation is repeated 5 times resulting in a total of 25 training/validation iterations. In order to eliminate the bias from individual steers, i.e., so that each steer has an equal contribution during model training, the training set was balanced. More precisely, each steer was represented with the same number of observations as the steer with the shortest observational period across all 12 individual animals. The remaining segments derived from steers with longer observational periods are under-sampled randomly, with the time-order of given observations remaining unchanged. Further, each feature is standardised, so that each feature time-series had zero-mean and unit-variance, to ensure that feature scales are comparable i.e.,:

(1) $$x_{i}^{\prime }=\frac{x_{i}-\mu_{i}}{\sigma_{i}},\;\forall i\in \left\{1,\cdots ,42\right\},$$

where $x_{i}$ and $x_{i}^{\prime }$ represent the original and standardised feature vectors, respectively, while $\mu_{i}$ and $\sigma_{i}$ refer to the mean and standard deviation of the corresponding feature prior to standardisation. Both $\mu_{i}$ and $\sigma_{i}$ are estimated on the training set and consequently each fold results in different normalisation parameters but those parameters are used for both training and validation sets.

Naturally, each steer spends varying amounts of time in each of the states and as a consequence resulting in an unequal number of observations per class; for that reason, a balanced accuracy is used to evaluate model performance;

(2) $$Balanced\;accuracy=\frac{TP}{P}+\frac{TN}{N}$$

Equation (2) relates to binary classification problems. In cases where more than 2 classes are present (as is the case in the study reported here), individual class estimations are required and the average balanced accuracy can be used to evaluate overall performance. True Positives (*TP*) represent the number of accurately detected observations of a certain class, while True Negatives (*TN*), the number of observations accurately detected as not belonging to that particular class. Variables *P* and *N* refer to the actual number of observations belonging to the class of interest and the actual number of observations of all other classes, respectively.

### 4.2. Feature Reduction

The process of dimensionality reduction i.e., the representation of high-dimensional data in a lower-dimensional feature space, not only mitigates the curse of dimensionality but also reduces the computing resource requirements, model training and inference times [24].

A reduction of data dimensionality can be performed through feature transformation, feature selection, or a combination of both, providing the intrinsic dimensionality (minimum number of parameters needed to account for the observed properties of the data [25]) of the original feature set. Feature transformation methods are a reconstruction process of the original features into a new feature set. However, it should be noted that these techniques do not reduce the number of features that need to be selected from the raw data but rather, re-project the original features onto a new domain. The goal of feature selection is to establish a subset of features, retaining those with a higher discriminatory power. The selection can be executed in a number of ways depending on the goal, available resources, and the target level of optimisation [26]. Feature selection methods are most commonly classified into three categories: filters, wrappers, and embedded methods (Figure 2). Filter methods estimate feature relevance based on a ranking function which observes input or input/output data and drops low-scoring features. Filter methods are computationally inexpensive and independent of classification model, and as such, need only to be executed once to obtain the most appropriate features, which can be subsequently used to create and evaluate classification models [27]; both the wrapper and embedded approaches require the training of the model. In particular, the wrapper method requires multiple training iterations for multiple feature combinations, increasing significantly the computational cost. Embedded methods are based on intrinsic properties of the classifier and performed during model training. Although the two approaches are based on an interaction between the extracted features and classification model, common drawbacks of the wrapper approach are a higher risk of over-fitting as well as consuming greater levels of computing resources compared to filter methods.

Here, two feature selection methods are evaluated, namely, the filter method based on the Mutual Information (MI) score and the wrapper method based on the Backward Feature Elimination (BFE) technique. Embedded approaches are not considered since feature ranking is not implicitly supported by other reported classification algorithms. MI is a statistical measure which estimates the dependence between different sets of data, the value of zero referring to completely independent sets, while higher values represent a higher dependency. In this particular case, the dependence between individual features and labels is analysed. The most applied approach for MI estimation assumes the partitioning of the datasets into bins of finite size. However, here, MI was estimated using the k-nearest neighbour method [28]. A comparison between binning and the nearest neighbour method, along with the following definition of the MI estimate between discrete and continuous datasets is given in [29];

(3) $$I\left(X,Y\right)=\psi \left(N\right)-\langle \psi \left(N_{x}\right)\rangle +\psi \left(k\right)-\langle \psi \left(m\right)\rangle$$

where ψ is the digamma function, while 〈·〉 denotes the average over all samples. $N_{x}$ represents the number of points per activity state and *m* refers to the number of neighbours from all states that lie within the defined distance determined by parameter *k*. Although larger values of *k* lead to lower statistical errors, excessively large values of *k* should be avoided since the resultant increase in systematic errors could potentially outweigh the decrease in statistical errors. The analysis conducted in [29] indicates that the nearest-neighbor estimator achieves good performance when the parameter *k* is set to low integer values (1≤k≤10), whereas the authors in [28] suggested a range between 2 and 4. For that reason, here, the number of nearest neighbours is set as k=3.

The second feature selection approach investigated is the wrapper method using BFE, reliant on the use of the classification model to execute feature selection. The approach typically starts by iteratively removing one or more features from the entire feature set $F=f_{1},\cdots ,f_{N}$, governed by the performance of the classification model on the selected subset of features. More precisely, the methodology follows the steps of the algorithm reported in Algorithm 1;
**Algorithm 1** Backward Feature Elimination procedure used to reduce features in blocks.  $F=f_{1},\cdots ,f_{N}$▹ Total features set  R=F▹ Remaining features set  *P*▹ Declare empty performance array  **while**
|R|>1
**do**
       **for** i← 1 to *R* **do**
          $S_{f}=R-f_{i}$▹ Select subset of features $S_{f}\subset F$          model.fit$\left(S_{f}\right)$▹ Train the model with $S_{f}$          P[i] = model.eval$\left(S_{f}\right)$▹ Compute model performance with $S_{f}$ features       **end for**
      $R=R\backslash \cup_{j=1}^{k}\left[R\backslash f_{\underset{j}{argmin}\left(P\right)}\right]$ where k≥1▹ Update remaining features by excluding low performing features  **end while**

## 5. Classification Algorithms

The effectiveness of each feature reduction technique is assessed through three classification algorithms, namely, Hidden Markov Model (HMM), Linear Discriminant Analysis (LDA), and Partial Least Squares Discriminant Analysis (PLS-DA). Note that both the LDA and PLS-DA perform feature transformation in accordance with the classifier operation inherently but do not reduce the number of inputs that must be computed from the raw data.

### 5.1. Hidden Markov Models

A probabilistic time-series model requires the definition of a joint distribution $p(X_{1},\ldots ,X_{T})$ where $X_{t}$ represents the features of a 90 s block in a sequence t∈1→T. The sequence has many entries with long-range correlations amongst subsequent observations. However, an independent specification of that many entries is impractical; therefore, simplifications are required. The main assumption underpinning Markov chains is that the current $X_{t}$ contains sufficient amount of information to predict future states i.e., that the influence of the recent past is more relevant than the influence of a more distant past [30]. A first order Markov chain is defined as follows:

(4) $$p\left(X_{1:T}\right)=p\left(X_{1}\right)\prod_{t=2}^{T}p\left(X_{t}|X_{t-1}\right)$$

where the conditional distribution $p(X_{t}|X_{t-1})$ for *K* states, can be written as a KxK transition matrix when $X_{t}\in \left\{1,\ldots ,K\right\}$ and the elements of the matrix represent the transition probabilities between states [31].

HMMs are an extension of Markov chains. Initially, for each observation $X_{t}$, a corresponding hidden variable $h_{t}$ is introduced, with $X_{t}$ dependent on $h_{t}$ through an emission probability $p(X_{t}|h_{t})$. A HMM is defined as;

(5) $$p\left(h_{1:T},X_{1:T}\right)=p\left(X_{1}|h_{1}\right)p\left(h_{1}\right)\prod_{t=2}^{T}p\left(X_{t}|h_{t}\right)p\left(h_{t}|h_{t-1}\right)$$

where $p(h_{1})$ is the initial probability. Here, emissions have been constructed through the Gaussian Mixture Model, a linear superposition of *K* Gaussian distributions defined as [32]:

(6) $$p\left(X\right)=\sum_{k=1}^{K}\pi_{k}N\left(x|\mu_{k},\Sigma_{k}\right)$$

where $N(x|\mu_{k},\Sigma_{k})$ denotes the Gaussian component with mean $\mu_{k}$ and co-variance $\Sigma_{k}$, and parameter $\pi_{k}$—known as mixing probability—such that $\pi_{k}\in \left[0,1\right]$ and $\sum_{k=1}^{K}\pi_{k}=1$.

The HMM parameters such as the transition matrix, emission matrix, and initial probability are optimised through an iterative procedure—the Expectation Maximisation (EM), also known as the Baum-Welch algorithm [33]. HMM optimisation requires the implementation of stopping criteria either in terms of the number of iterations or error tolerance. A thorough procedure is followed to estimate the optimal stopping criterion; a tolerance of 0.04 yielded the maximum average performance within an average execution time of 12 s per fold, utilising all 42 features.

HMMs can also consider the temporal behaviour of the signal, taking into account a transition probability between states e.g., from ‘eating’ to ‘rumination’, the main motivation for the evaluation of their potential performance for cattle behavior classification.

### 5.2. Linear Discriminant Analysis

Fitting joint probability density function models to determine a decision boundary can be problematic in data with high dimensions; hence the need to reduce the input data dimensionality [31]. Unlike HMM, LDA is a supervised technique, making use of labels alongside the features in the dataset. LDA searches the dimensions in the underlying space that maximise the distance between the means of different states (inter-class variance) and minimises the variation within each category (intra-class variance) [34]. More formally, LDA creates a linear combination of input features with the goal to maximise the ratio $\frac{\det|S_{b}|}{\det|S_{w}|}$, where $S_{b}$ and $S_{w}$ are the intra-class and inter-class scatter matrices respectively as defined in [35]. The disadvantage is that the approach fits a Gaussian density to each class, assuming that all classes share the same co-variance matrix. Furthermore, LDA projects the original space to a lower dimensional space which is limited to ≤K−1 dimensions (where *K* is the number of classes), regardless of the dimensionality of the input.

### 5.3. Partial Least Squares Discriminant Analysis

A Partial Least Squares algorithm is developed initially as a regression technique and extended subsequently for classification tasks and its discriminant form (PLS-DA) [36]. Similar to LDA, a PLS-DA is a supervised technique that combines dimensionality reduction and discriminant analysis. However, unlike LDA, PLS-DA does not assume that the input data fits a single Gaussian distribution. PLS aims to maximise the variance of the response variables (labels) explained by the explanatory ones (features) [37]. The optimisation of the Nonlinear Iterative Partial Least Squares (NIPALS) algorithm involves computing the singular vectors of the cross co-variance matrix. A tolerance of $10^{-6}$ and 500 iterations are used as stopping criteria for the optimisation, consuming an average execution time of 0.73 s per fold, for all 42 features re-projected into 42 dimensions. The formal definition of PLS-DA used is described in [38].

Given that PLS regression analyses generate a continuum of predicted values, the definition and application of a decision rule is required to translate the predicted values in one of the corresponding classes. The most commonly used reported decision rule is a class assignment based on the maximum positive value [36] of the predicted output variables, henceforth used within this analysis.

## 6. Performance Evaluation

A systematic evaluation of the performance of the classification of cattle states as a function of different combinations of dimensionality reduction and classification techniques is carried out. Dimensionality reduction is implemented using the two feature selection techniques detailed thus far, namely, the filter method based on the MI score and the wrapper method based on the BFE, with three classification algorithms viz. HMM, LDA, and PLS-DA. A grid search to optimise the number of features that optimally discriminate between states is performed for each combination. The number of features decreases gradually starting from the full dataset containing 42 features. The reduction in dimensionality is executed in nine steps as reducing one feature at a time is computationally prohibitive; as a consequence, the number of features is decreased in steps of five until a single feature is reached. Feature transformation methods are not considered as they do not reduce the number of features from the raw data.

The HMM is implemented using *hmmlearn* Python framework (https://github.com/hmmlearn/hmmlearn, accessed on 16 February 2022), while LDA and PLS-DA are implemented utilising the *scikit-learn* Python library [39]. The BFE algorithm was implemented in Python as described in Algorithm 1.

Due to the stochastic nature of the training process, many folds and repetitions may result in elimination of different subsets of features. Thus, a ranking methodology is required to reach consensus on feature importance. For MI, a simple feature ranking process computes the MI feature score for all folds and repetitions in the training set and subsequently utilises the average MI score of each feature as a proxy of importance. The dimensionality of the data is then reduced by eliminating a pre-defined number of the least important features (five in this case). On the other hand, BFE utilises a classification algorithm and thus feature importance can be inferred based on classification performance on the validation set. Here, multiple training/validation stages are performed by excluding one feature at a time for all folds and repetitions; the process yields 25 balanced accuracy results for each feature (five folds with five repetitions). The average balance accuracy is then used to determine the feature importance rank with the lowest average rank features from the pre-defined number eliminated.

**Figure 3:**
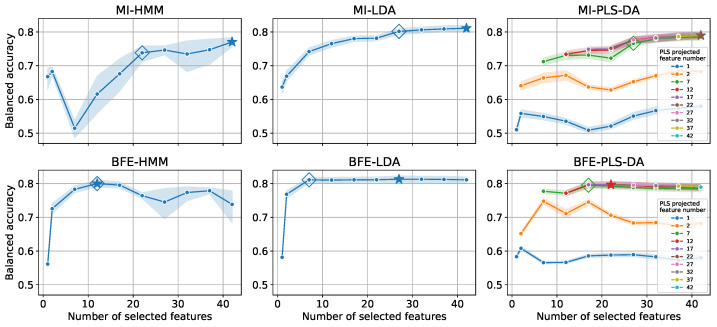
Balanced Accuracy for HMM, LDA and PLS-DA classification algorithms for two feature selection methodologies; MI and BFE for varying number of selected features. For PLS-DA the number of re-projected feature dimensions were varied to explore sensitivity of the hyper-parameter. The ⋆ denotes models with maximum balanced accuracy performance, while the ⋄ denotes models that were manually selected and balance the trade-off between balanced accuracy and time complexity.

The average balanced accuracy and the 95% confidence interval (the Confidence Interval is computed with boot-strapping [40]) on the validation dataset for all combinations, is shown in Figure 3 for varying degrees of reduction. Note that the models with the maximum validation performance are highlighted by a star (⋆) and the diamond (⋄) represents models that exhibit almost identical performance with the minimum number of features (hence decreasing computational complexity). The ‘⋄’ locations are selected manually, taking into consideration the knee point for the line graphs.

The top row plots relate to MI feature selection. Since MI only utilises input and output data for the scoring and not a model, all resultant features at each reduction step are identical for all classification algorithms. As the number of selected features decreases, the balanced accuracy drops as well, indicating that MI is not effective in identifying redundant features for all classification algorithms. Although MI is an efficient statistical measure to estimate the dependence between individual features and output, correlations between features are not considered. As a result, the subset of features that survive the dimensionality reduction have high Mutual Information between input and output but are highly correlated with each other without providing additional discriminatory power. The performance of the reduction for MI-LDA and MI-PLS-DA is not as steep as MI-HMM, since an inherent re-projection of the input feature space onto a lower dimensional one is performed by eliminating redundant information providing a higher level of robustness against the over-fitting. For PLS-DA the desired size of the lower dimensional space after re-projection is a model hyper-parameter and in order to evaluate the performance, a number of models were trained for a multiple number of re-projected dimensions. In particular, each line in the MI-PLS-DA (and BFE-PLS-DA) corresponds to the dimensionality of the final projected space. For instance, the red line corresponds to balanced accuracy as a function of the number of raw features selected, all re-projected to 12 dimensions; consequently, the line does not extend to below 12 on the horizontal axis.

Similarly, the bottom row of the figure, presents the results using BFE. In general, the performance of all models is higher than the corresponding performance with MI, even for a significantly lower number of selected features, attributable to a more structured feature selection methodology. Note that for 42 selected features, the average balanced accuracy obtained through the five folds and five repetitions is lower for BFE-HMM compared to MI-HMM. This is caused by the EM algorithm which is gradient-based and gets stuck in local minima [31] at convergence. In turn creates outlier results with a low balanced accuracy (~0.3), also evident by a wider range of the confidence interval. For the band 22–32 of the selected features, the balanced accuracy of BFE-HMM drops owing to the greedy nature of the feature elimination, i.e., decreasing the features in steps of five without reevaluating prior reductions. BFE-LDA and BFE-PLS-DA are more robust to feature reduction, maintaining performance due to their inherent feature transformation. The performance with 7 features is nearly equal to the maximum performance obtained for 27 features for BFE-LDA. For BFE-PLS-DA, the maximum performance is achieved for 22 features which are re-projected to 12; however, the performance is almost identical to 17 features re-projected to 7. The re-projection reduces the computational complexity of the inference, hence, in the manual selection of the ‘⋄’ locations we favoured the re-projections onto lower dimensions.

Table 1 presents the balanced accuracy on the validation set and computation complexity in terms of feature extraction and inference times for all combinations of models and feature selection approaches. In this study, all analyses are conducted on commodity hardware; 64-bit Intel i9 7960x 2.8 GHz 16 cores 128 GB RAM for the purposes of evaluation; however, it is expected that the relative performance differences will translate to low-power resource constraint processors. Furthermore, Table 1 presents the number of raw features that need to be computed for each methodology. For instance, the LDA classification model with maximum performance (‘⋆’) obtained through BFE technique, requires the extraction of 27 features consuming a computation time of 38.05 ± 3.89 ms and 0.05 ± 0.01 ms for inference, and achieving an average balanced accuracy of 0.81 on the validation dataset. The corresponding BFE-LDA ‘⋄’ model requires the computation of 7 raw features which on average, consumes 1.83 ± 1.00 ms for extraction and 0.05 ± 0.01 ms for inference, reducing the total time required ~20 times without loss in balanced accuracy, in contrast to BFE-LDA ‘⋆’.

The computational performance difference between a desktop machine and a low-power MCU can be estimated by using the floating point performance as a proxy for the mathematical operations required for feature extraction and inference. An ARM Cortex-M4 [41] requires 9 CPU cycles to complete an FP32 Multiply-and-accumulate (MACC) operation, whereas an Intel i9 can complete 2 MACC per cycle [42]. When the clock speed of each chip is considered, the difference in computational capability between this CPU and an ARM Cortex-M4 such as STM32L476RG [43], is a factor in the region of 1000, with timings scaling appropriately. This factor reduction in computation performance would result in total time complexity increasing; ranging from 1.96s (BFE-LDA ‘⋄’) to 95s (MI-HMM ‘⋆’). Hence, the model with the lowest time complexity is still comfortably within a time complexity for deployment to a MCU.

**Figure 4:**
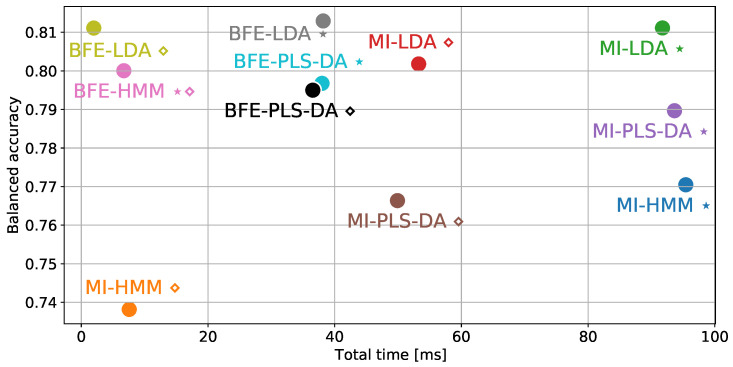
Graphical comparison of dimensionality reduction and classification algorithms, in terms of time complexity and performance.

In addition to the information provided within Table 1, Figure 4 presents a graphical comparison between models and illustrates the trade-off between model performance and complexity, through the average validation balanced accuracy and average total processing time, respectively. Although BFE-LDA ‘⋆’ is highest performing, it is evident that BFE-LDA ‘⋄’ achieves almost identical validation performance at significantly lower computational complexity, requiring only 1.83 ± 1.00 ms for feature extraction and 0.05 ± 0.01 ms for inference. Therefore, the BFE-LDA ‘⋄’ would be selected for implementation in resource constrained hardware and is the model considered in the remainder of the analysis.

**Figure 5:**
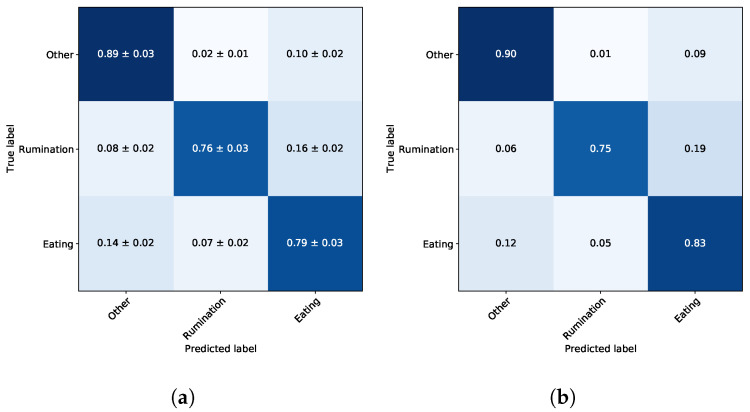
Confusion matrices for the selected classification model based on a LDA utilising features selected through BFE that yielded the best trade-off between model performance and complexity—BFE-LDA ‘⋄’. (**a**) Validation dataset. (**b**) Test dataset.

The mean validation performance of BFE-LDA ‘⋄’, in terms of the weighted performance metrics of balanced accuracy, precision, and recall are 0.81, 0.85 and 0.82, respectively. The average validation confusion matrix, along with the standard deviations, is shown in Figure 5a. The confusion matrix indicates the normalised individual performance for all states, e.g., the normalised True Positive performance for ‘eating’ is 0.79 ± 0.03, while ‘rumination’ is mis-classified as ‘eating’ 0.16 ± 0.02; in all cases, the standard deviation is below 0.03. The BFE-LDA ‘⋄’ model with the highest performance on the validation set is selected for evaluation of performance on the test set.

**Figure 6:**
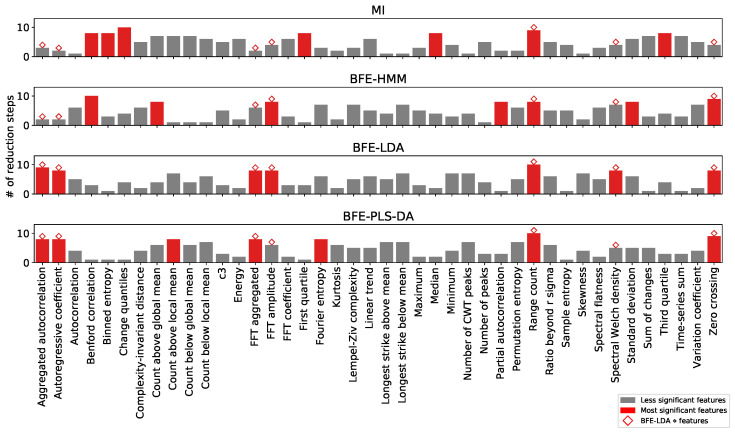
Number of reduction steps the features survived for MI and BFE selection methods. The ⋄ annotations represent the seven features selected by BFE-LDA ‘⋄’ and red bars the seven features that survived most reductions for each feature selection algorithm.

**Figure 7:**
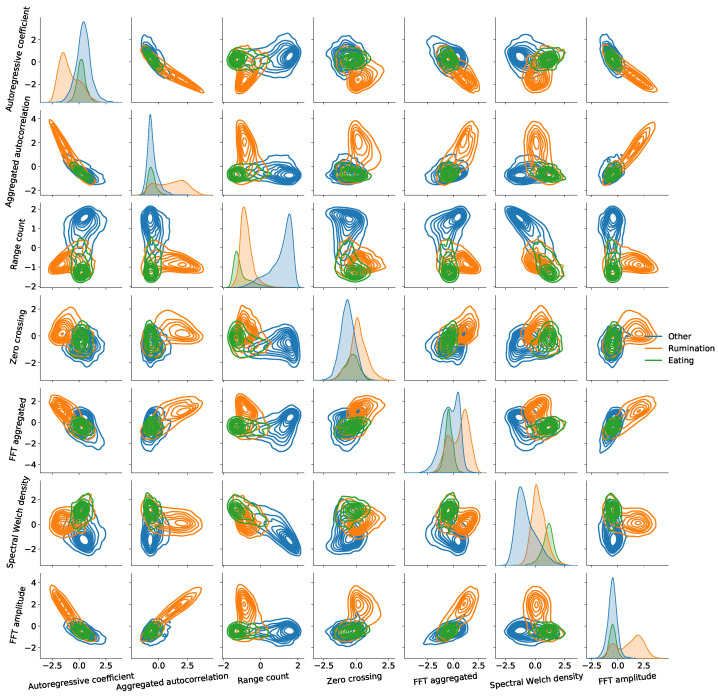
Joint distribution of feature pairs selected by BFE-LDA ‘⋄’ with class annotations provided by the truthing data. Note that the diagonal plots are the univariate distributions of each feature.

Further insight arising from the feature selection comparisons can be obtained through the visualisation of the feature importance results for each of the selected combinations of feature reduction method and classification algorithm. Figure 6 shows the feature importance based on the number of reduction steps. Unlike the BFE, MI based feature selection does not depend on the classification algorithm and hence only one graph is shown for all models. Since BFE-LDA ‘⋄’ yields the optimum trade-off between performance and time complexity, it is used as a base line for comparison. The seven features selected by BFE-LDA ‘⋄’ are annotated in all graphs with a ‘⋄’ and the seven most significant features of each approach are annotated in ‘red’. To get consensus between feature selection algorithms, all red bars will be accompanied by a ‘⋄’ annotation. It is clear that BFE-PLS-DA and BFE-LDA have the highest agreement in terms of feature significance and swaps the *FFT amplitude* and *Spectral Welch density* with *Count above global mean* and *Fourier entropy*. Nevertheless, the excluded features have considerable importance. On the other hand, MI and BFE-HMM only agree on ranking feature importance for one and three features respectively. Finally, visualisation of the joint distribution pairs of the seven most important feature combinations selected by BFE-LDA and annotated based on truthing data is presented in Figure 7. Evident is the strength of the knowledge-specific feature *FFT amplitude* which represent the amplitude in the spectral range between 2–4 Hz, aligned with a priori knowledge valuable in guiding the discrimination of the ‘rumination’ class. The *Range count* feature provides a high separation for the ‘other’ class, while for the ‘eating’ class a combination of features are likely to be required. It should be noted that the second knowledge-specific feature *Spectral flatness* only survived 5 reduction steps for BFE-LDA (see Figure 6) inferring that other generic features of higher importance exist.

The average weighted metrics for the BFE-LDA ‘⋄’ in the test set are 0.83, 0.88 and 0.83 for balanced accuracy, precision, and recall, respectively. The metrics were also recorded for individual results per steer on the test set shown in Table 2. Furthermore, the performance of individual classes, ‘eating’ and ‘rumination’, are 0.90 and 0.90 in terms of a precision and 0.86 and 0.90 in terms of recall, respectively.

The normalised confusion matrix on the test set is shown in Figure 5b. Although every attempt is made to mount collars in identical positions around the necks of individual animals, differences in the anatomy and dynamic motion of the animal result in the collars shifting and rotating which results in variations of the accelerometer output, in turn establishing another source of noise that is likely to compromise the accuracy of the classification. It is also clear that the confusion between ‘eating’ and ‘rumination’ is the greatest, as those two states are characterised by similar jaw motions. Given the similarity in these jaw motion patterns, some confusion is to be expected, especially during transition periods. The degree of confusion between other states is lower. In order to place these results in context, assuming the average time spent ruminating is around 400 min per day, an increase in sensitivity of 1% would represent an increase of ~4 min of time spent ruminating.

## 7. Conclusions

Autonomous sensor-based cattle behaviour monitoring systems have grown in importance over the recent past, as an enhancement to traditional visual methods which are both time-consuming and labour-intensive. Systems such as neck-mounted collars monitor dairy and beef cattle continuously, providing a mapping of key behaviours at an individual animal level automatically, the basis for a decision support capability that informs on interventions that enhance the efficiency of current on-farm practices. Here, a novel approach to the development of behaviour classification algorithms, founded on a systematic approach to reducing the dimensionality of the data is reported. Two feature selection techniques based on a MI score and BFE techniques are applied to both knowledge-specific and generic time-series features extracted from raw accelerometer data. A total of 42 features are extracted from raw accelerometer signals as the starting point, subsequently reduced to 7 with the goal of optimising the degree of discrimination between three key cattle behaviours—‘eating’, ‘rumination’ and ‘other’. The rationale underpinning the selection of the combination of reduction technique and classification algorithm framework is presented and a systematic evaluation of performance provided. The trade-off between model performance, computational complexity and memory footprint is explored. Results show that proposed Backward Feature Elimination to execute on feature selection provides features with higher discriminatory power at the expense of higher computational complexity. Post feature selection, Linear Discriminant Analysis yields a classification model with an overall balanced accuracy of 0.83 and is the most efficient from all of feature reduction/algorithm combinations considered in the paper in terms of implementation in operational settings. In particular, the best combination requires 1.83 ± 1.00 ms to perform feature extraction with 0.05 ± 0.01 ms for inference, thus permitting model deployment within the computation and memory restrictions imposed by operational settings. Evidence is provided that the proposed methodology represents a viable option in the evolution of low-cost neck-mounted accelerometer-equipped collars within precision livestock farming applications.

The dataset generated in this study, including raw data and ground truth annotations from 18 steers, has been made publicly available to stimulate the community to develop new models and facilitate direct comparisons between them (doi:10.5281/zenodo.4064802). Further research should aim to expand in larger trials with more animals and longer observation periods to increase the confidence of behavioural classification and identify novel value-added services.

## Figures and Tables

**Table 1:** Comparison of model performance and time complexity for MI and BFE feature selection approaches for HMM, LDA and PLS-DA classification algorithms. The ⋆ models achieve maximum balanced accuracy performance, while the ⋄ models are those that are manually selected and balance the trade-off between balanced accuracy and time complexity.

Feature Selection	Classification	# of Input	Balanced	Time Complexity [ms]		
Technique	Method	Features	Accuracy	Extraction	Inference	Total
MI	HMM ⋆	42	0.77	94.68±9.84	0.64±0.17	95.42±9.91
	HMM ⋄	22	0.74	6.99±0.89	0.51±0.03	7.57±0.91
	LDA ⋆	42	0.81	91.60±6.65	0.05±0.01	91.74±6.66
	LDA ⋄	27	0.80	53.08±1.21	0.05±0.01	53.25±1.22
	PLS-DA ⋆ Projected to 22 features	42	0.79	93.50±5.94	0.06±0.01	93.64±5.95
	PLS-DA ⋄ Projected to 7 features	27	0.77	49.78±3.06	0.04±0.01	49.90±3.06
BFE	HMM ⋆⋄	12	0.80	6.12±0.53	0.53±0.03	6.71±0.55
	LDA ⋆	27	0.81	38.05±3.89	0.05±0.01	38.18±3.89
	**LDA**⋄	**7**	**0.81**	1.83±1.00	0.05±0.01	1.96±1.01
	PLS-DA ⋆ Projected to 12 features	22	0.80	37.86±4.58	0.06±0.01	37.99±4.58
	PLS-DA ⋄ Projected to 7 features	17	0.79	36.40±4.24	0.05±0.01	36.54±4.25

**Table 2:** Individual classification performance per steer in terms of weighted performance metrics on the test set.

Test Steer	Balanced Accuracy	Precision	Recall
#1	0.82	0.86	0.85
#2	0.86	0.90	0.87
#3	0.80	0.89	0.79
Average	0.83 ± 0.03	0.88 ± 0.02	0.83 ± 0.04

## Data Availability

The dataset is publicly available at https://www.doi.org/10.5281/zenodo.4064802 (accessed on February 2022).

## Appendix A

**Table A1:** Brief description of generic and knowledge-specific time-series features. All the features used within the analysis are derived using the *tsfresh* Python package [23] with the exception of *FFT amplitude* and *Spectral flatness*.

Features	Definition
Aggregated autocorrelation	Standard deviation of autocorrelation function over a range of different lag values
Autoregressive coefficient	Coefficient of the unconditional maximum likelihood of an autoregressive process
Autocorrelation	$\frac{1}{\left(n-\mathrm{lag}\right)\sigma^{2}}\sum_{i=1}^{n-\mathrm{lag}}\left(x_{i}-\mu \right)\left(x_{i+\mathrm{lag}}-\mu \right)$
Benford correlation	Correlation of the time-series first digit distribution with N-B Law distribution
Binned entropy ${}^{†}$	$-\sum_{i=0}^{min(n_{bins},n)}p_{i}\log p_{i}*1_{\left(p_{i}>0\right)}$
Change quantiles	Standard deviation of changes of the time-series within the first and third quartile range
Complexity-invariant distance	$\sqrt{\sum_{i=1}^{n-1}{\left(x_{i}-x_{i+1}\right)}^{2}}$
Count above global mean	Number of observations higher than the mean value estimated on the training set
Count above local mean	Number of observations higher than the time-series mean
Count below global mean	Number of observations lower than the mean value estimated on the training set
Count below local mean	Number of observations lower than the time-series mean
c3	$\frac{1}{n-2\mathrm{lag}}\sum_{i=1}^{n-2\mathrm{lag}}\left(x_{i+2\mathrm{lag}}*x_{i+\mathrm{lag}}*x_{i}\right)$
Energy	$\sum_{i=1}^{n}x_{i}^{2}$
FFT aggregated	Kurtosis of the absolute Fourier transform spectrum
FFT amplitude	Maximum of FFT magnitudes between 2 and 4 Hz
FFT coefficient	Sum of the FFT magnitudes between 2 and 4 Hz
First quartile	The value surpassed by exactly 25% of the time-series data points
Fourier entropy	Binned entropy of the time-series power spectral density
Kurtosis	Difference between the tails of analysed distribution and tails of a normal distribution
Lempel-Ziv complexity	Complexity estimate based on the Lempel-Ziv compression algorithm
Linear trend	Standard error of the estimated linear regression gradient
Longest strike above mean	Length of the longest sequence in time-series higher than its mean value
Longest strike below mean	Length of the longest sequence in time-series lower than its mean value
Maximum	The highest value in time-series
Median	The value surpassed by exactly 50% of the time-series data points
Minimum	The lowest value in time-series.
Number of CWT peaks	Number of peaks within ricker wavelet smoothed time-series
Number of peaks	Number of observations with a value higher than *n* neighbouring observations
Partial autocorrelation	$\frac{cov(x_{t},x_{t-\mathrm{lag}}|x_{t-1},\ldots ,x_{t-\mathrm{lag}+1})}{\sqrt{var\left(x_{t}|x_{t-1},\ldots ,x_{t-\mathrm{lag}+1}\right)var\left(x_{t-\mathrm{lag}}|x_{t-1},\ldots ,x_{t-\mathrm{lag}+1}\right)}}$
Permutation entropy	Entropy of ordering permutations occurring in fixed-length time-series window chunks
Range count	Number of observations between the first and the third time-series quartile
Ratio beyond *r* sigma	Percentage of observations diverging from the mean by more than *r* standard deviations
Sample entropy	Negative logarithm of the conditional probability that two sequences remain similar
Skewness	Distortion or asymmetry that deviates from the normal distribution
Spectral flatness	Ratio between geometric and arithmetic mean of the power spectrum
Spectral Welch density	Power spectral density estimation using the Welch method at a certain frequency
Standard deviation	$\sqrt{\frac{1}{n}\sum_{i=1}^{n}{\left(x_{i}-\mu \right)}^{2}}$
Sum of changes	$\sum_{i=1}^{n-1}\left|x_{i+1}-x_{i}\right|$
Third quartile	The value surpassed by exactly 75% of the time-series data points
Time-series sum	$\sum_{i=1}^{n}x_{i}$
Variation coefficient	Relative standard deviation, i.e., ratio of the standard deviation to the mean
Zero crossing	Number of points where time-series signal crosses a zero value

† where *p*_*i*_ indicates percentage of samples falling into the given bin.
